# Simple chest closure of open window thoracostomy for postpneumonectomy empyema: a case report

**DOI:** 10.1186/s40792-019-0612-y

**Published:** 2019-04-05

**Authors:** Tetsuya Fukui, Tadashi Matsukura, Yusuke Wakatsuki, Satoko Yamawaki

**Affiliations:** 1 Department of General Thoracic Surgery, Japanese Red Cross Fukui Hospital, 2-4-1 Tsukimi Fukui, Fukui, 918-8501 Japan; 2 Department of Plastic Surgery, Japanese Red Cross Fukui Hospital, Fukui, 2-4-1 Tsukimi Fukui 918-8501 Japan

**Keywords:** Postpneumonectomy empyema, Bronchopleural fistulae, Open window thoracostomy, Omentopexy, Chest closure

## Abstract

**Background:**

Management of postpneumonectomy empyema requires comprehensive strategies, especially when the condition is associated with large bronchopleural fistulae. We report a case involving the simple chest closure of open window thoracostomy with remaining residual space.

**Case presentation:**

We performed open window thoracostomy for empyema with a huge bronchial stump dehiscence after right pneumonectomy for a large lung cancer. We definitively closed the chest window infected with chronic persistent *Pseudomonas aeruginosa* via a simple chest closure technique with the remaining residual space, after repairing the bronchial dehiscence using an omental flap and the appearance of healthy granulation tissue throughout the cavity. The patient died of recurrent cancer 10 months after the definitive chest closure. Until the patient died, there were no symptoms or signs suggestive of recurrent empyema.

**Conclusion:**

This simple chest closure technique allows “silent empyema” to be observed carefully, is less invasive, and can even be applied to cases of recurrent cancer.

## Background

The management of postpneumonectomy empyema (PPE) requires comprehensive strategies, especially when it is associated with large bronchopleural fistulae (BPF) or advanced pyothorax. We report a case involving open window thoracostomy (OWT) for PPE with BPF, which was definitively closed via a simple chest closure technique with the remaining residual space, following the repair of BPF and the appearance of healthy granulation tissue throughout the cavity.

## Case presentation

**Fig. 1:**
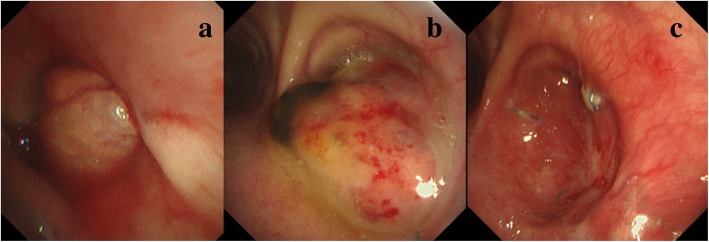
Bronchoscopy demonstrates definitive closure of the right main bronchus dehiscence. The operative day of the repair with the omentum (**a**). Postoperative day 7 (**b**). At 4 months after the omentopexy (**c**)

**Fig. 2:**
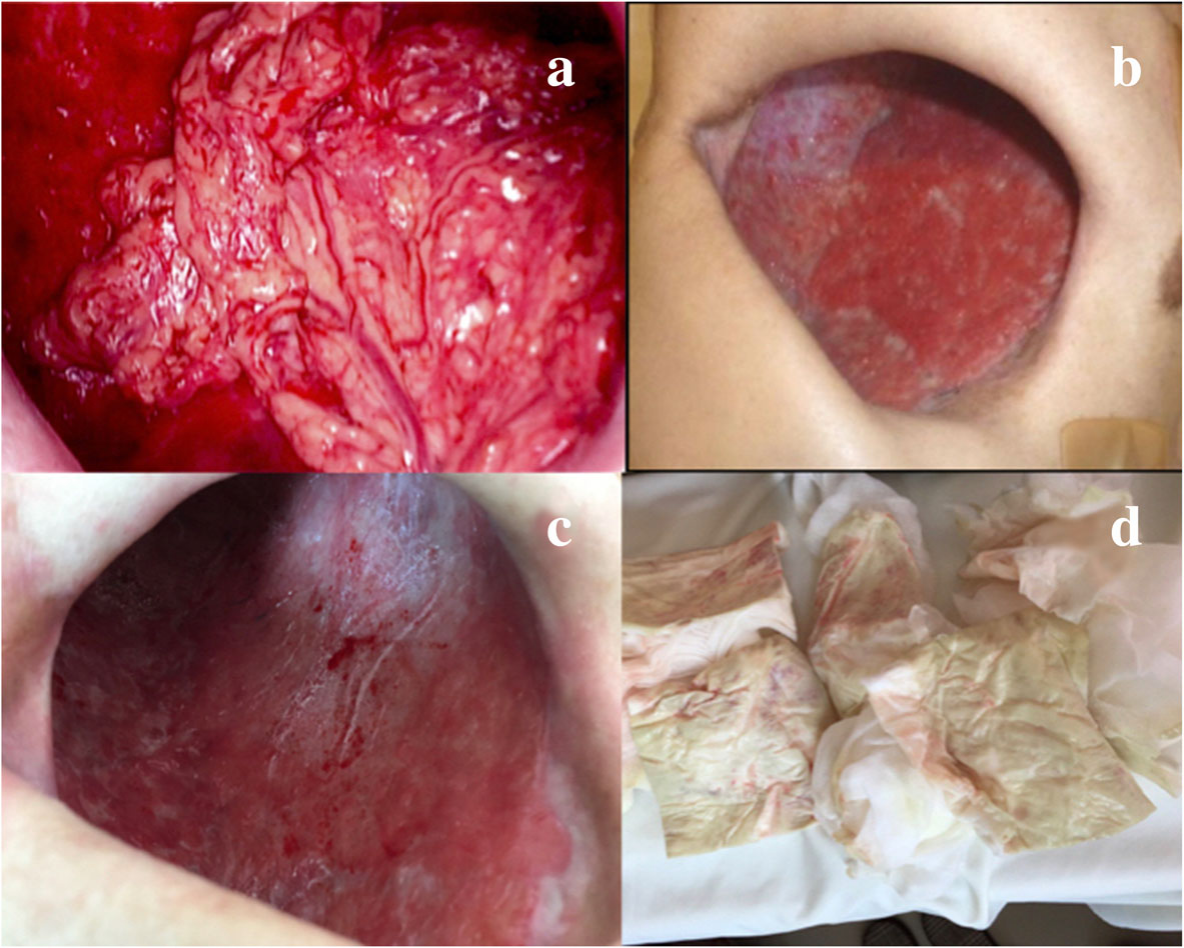
Bronchopleural fistula closure promoted the growth of healthy thick granulation tissue covering the whole cavity, thereby reducing the cavity size. Postoperative day 1 (**a**). At 2 and 4 months after the omentopexy (**b**, **c**). The bacteria have not been eliminated completely from the cavity, and a small amount of yellowish exudate containing *Pseudomonas aeruginosa* is observed (**d**)

**Fig. 3:**
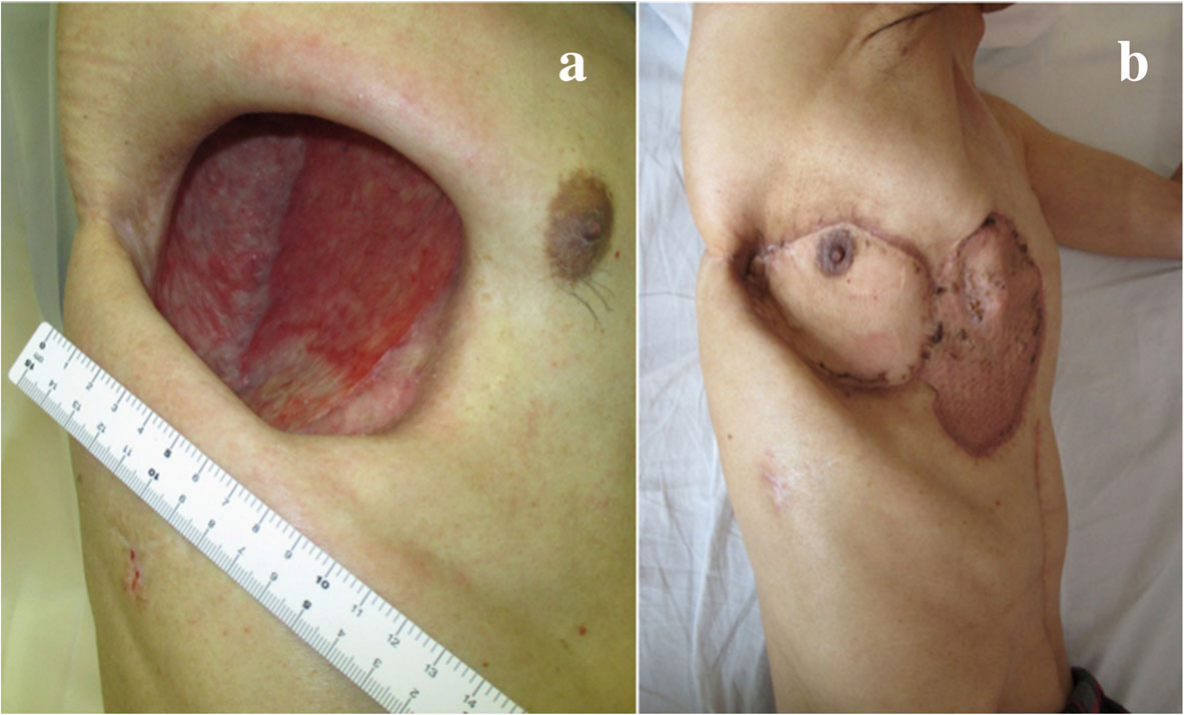
The window is simply closed with a pectoralis major flap without obliteration of the cavity (**a**). Split-thickness skin graft obtained from the thigh covers the area where the pectoralis major was harvested (**b**)

**Fig. 4:**
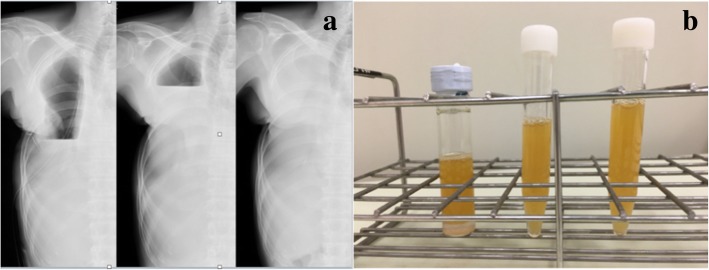
Following the simple chest closure, the residual cavity was gradually filled completely with pleural effusion without any symptoms of infection (**a**). Thoracentesis demonstrated slightly cloudy and yellowish pleural effusion, and *P. aeruginosa* was cultured from the fluid (**b**)

A 71-year-old man was referred to our center owing to the presence of a large right lung cancer, which was 18 cm in size and classified as clinical stage T4N0M0). The patient had parathyroid hormone-related peptide-mediated hypercalcemia and obstructive pneumonia. Following the treatment of hypercalcemia and pneumonia, we performed right pneumonectomy via posterolateral thoracotomy by dividing the latissimus muscle and a part of the serratus anterior muscle. The bronchial stump was covered with a pedicled intercostal muscle flap, achieving pathological curative resection. Pathological findings demonstrated acinar-predominant adenocarcinoma with wild-type *EGFR* and *ALK* and low PD-L1 expression—tumor proportion score (< 1%); the pathological stage was the same. His postoperative course was uneventful, and he was discharged on postoperative day (POD) 12. However, he was re-admitted to our center due to respiratory failure and severe sepsis on POD 30 owing to PPE combined with BPF. We performed thoracotomy debridement immediately, which demonstrated foul-smelling pus filling the cavity, intercostal muscle flap necrosis, and bronchial stump dehiscence 20 mm in diameter. Considering his poor general condition, we gave up primary closure of the BPF and performed OWT. The blood and thoracic pus cultures revealed *Streptococcus anginosus* and anaerobic gram-negative rods; therefore, the patient was treated with carbapenem for 2 weeks. After performing OWT, the color of the thoracic cavity wall and pleural effusions gradually turned spotty blue and green, and *P. aeruginosa* was cultured from the gauze after 1 month. Given the patient’s improved condition, we performed several debridement procedures for sterilization, and a thin granulation tissue covered the whole cavity, but *P. aeruginosa* was still present. Eight months after the fenestration, we closed the BPF with the pedicled omental flap. Laparotomy was performed to expose the omentum, which was dissected and brought into the pleural cavity through an incision in the diaphragm. Separation of the bronchial stump from the mediastinum and direct closure of the fistula seemed to be unsuccessful; thus, we used the vascularized omental flap as a patch and placed it to the edges of the fistula. The omentum was placed on the stump to cover the fistula and fixed with interrupted 4–0 monofilament sutures to achieve airtight closure. We only administered cefazoline to prevent surgical site infection, but not covering *P. aeruginosa*. The patient did not experience abdominal complications or adverse events caused by *P. aeruginosa* postoperatively. We continued to change the dressing daily through fenestration with bared omentum, which soon became granulated. On POD 3, air leakage from the stump occurred, which was detected by the sound of air flow and vibration of the omentum. It was managed by granulation on POD 5. The BPF was closed definitively using the well-vascularized omental flap (Fig. 1a–c). BPF closure promoted the accelerated growth of the healthy thick granulation tissue covering the whole cavity, thereby reducing the cavity size (Fig. 2a–c). We could not completely eliminate the bacteria from the cavity, and a small amount of yellowish exudate still contained *P. aeruginosa* (Fig. 2d). While considering the timing of definitive chest closure, cancer recurrences were detected in the brain and contralateral lung at 4 months after the bronchial closure. Brain metastasis was treated with radiotherapy, and the lung nodule was only observed closely. We decided to proceed with the definitive chest closure. We explained the risk and benefit of thoracostomy closure to the patient, who was eager to return to his normal activities of daily living. At 5 months after the bronchial closure, we simply closed the window using a pectoralis major flap (Fig. 3a) without performing complete obliteration of the cavity, such as thoracomyoplasty or antibiotic filling. Split-thickness skin graft obtained from the thigh covered the area where the pectoralis major was harvested (Fig. 3b). Cefazoline was administered intravenously to prevent surgical site infection, but not covering *P. aeruginosa*. The postoperative course was uneventful, and the residual cavity was gradually filled completely with pleural effusion but without any symptoms of infection (Fig. 4a). Thoracentesis performed at 3 months after chest closure demonstrated slightly cloudy and yellowish pleural effusion, and *P. aeruginosa* was cultured from the fluid (Fig. 4b). The pleural cavity seemed to contain a “silent empyema.” He was finally discharged and returned home at 15 months after the first pneumonectomy. The patient chose to receive supportive care. He died at 24 months after the pneumonectomy (10 months after the definitive chest closure) owing to recurrent cancer in the contralateral lung. Until he died, there were no symptoms or signs suggestive of recurrent empyema. Patient consent was obtained for the publication of this report.

## Discussion

Management of advanced infected empyema with large fistulae requires comprehensive treatment approaches. These strategies are challenging, consisting of the management of BPF and pleural cavity, control of infection, and optimization of general conditions [1–5]. The OWT was simply closed, and the residual space was gradually filled completely with pleural effusion, following the fenestration, sterilization, repair of bronchial dehiscence using the omental flap, and the appearance of healthy granulation tissue throughout the cavity.

Adequate and immediate pleural drainage is essential in PPE with BPF to prevent aspiration pneumonia and to control infection. OWT is the ideal method for the initial treatment of this condition [1, 6, 7]. Recently, rethoracotomy, debridement, and simultaneous bronchial closure with extraskeletal muscle flap, followed by negative wound pressure therapy and repeated thoracotomy for gauze change to accelerate the healing process, have been reported to be effective for PPE with BPF [8–10]. In this case, since the patient was fragile, had his thin latissimus muscle and a part of the serratus anterior muscle divided, had large stump opening, and had severe empyema, we considered that fenestration was appropriate. He had only mild respiratory failure with tight gauze packing to cover the BPF.

The optimal timing for BPF closure depends on whether the patient is in a stable condition and whether the cavity is clean [11, 12]. In this case, the presence of healthy granulation tissue throughout the cavity signified the appropriate timing for the procedure. Closure of the BPF is not complete without coverage of a vascularized tissue flap [11, 12]. Moreover, the utility and acceptable incidence of abdominal complications with omentoplasty have been reported [8, 11, 12]. When direct closure of the BPF is not possible, the opening can be closed with a pedicle flap of the omentum or muscle used as a patch [3, 8–11]. In this case, the well-vascularized omental flap, which was sewn to the edges of the bronchial fistula to avoid separation of the stump from the mediastinum or direct closure, definitively closed the bronchial dehiscence. Exposing the omentum pedicle flap through the diaphragm during the treatment of empyema with OWT did not cause any abdominal complications. The raw omentum was completely covered with granulation tissue after a week. BPF closure with the omental flap promoted healthy granulation tissue coverage and sterilization throughout the cavity.

The optimal timing of definitive chest closure depends on the condition of the pleural cavity. As mentioned above, the healthy granulation tissues covering the whole cavity represent a clean cavity and it is more considered for the timing of definitive chest closure, rather than the negative microbiologic culture results [3, 5, 6, 10–12]. BPF closure is also essential for the definitive chest closure. If the closure of the BPF is secure, the cavity can be obliterated at the time of BPF repair [11–13]. Definitive chest closure can be performed in a variety of ways, such as intrathoracic flap transposition, thoracoplasty, myothoracoplasty, and Clagett procedure (i.e., obliteration of the pleural cavity filled with antibiotic solution) [1–14]. In this case, we could not eliminate *P. aeruginosa* completely from the cavity, but the BPF closure with omentum promoted the healthy and thick granulation tissue growth, thereby reducing the cavity size. The patient had air leakage between PODs 3 and 5 after BPF closure, which was resolved by granulation healing. Hence, we consider that it is possible to avoid simultaneous BPF and chest closure procedures. For chest closure, we closed the thoracostomy with a “simple chest closure” technique utilizing a pedicled muscle flap, which retained the residual cavity inside. After the chest closure, the residual space was gradually filled completely with pleural effusion, containing *P. aeruginosa*. The pleural cavity seemed to contain a “silent empyema,” and was completely isolated from the living organism; thus, the patient did not develop any symptoms of infection. The reasons why the residual cavity was allowed to remain were as follows: (1) the patient was thin and lacked viable muscles, which were damaged by previous operations, (2) less invasive surgery was preferable for cancer recurrence; and (3) we considered whether the antibiotic solution administered in the cavity can completely eradicate persistent *P. aeruginosa*, and we were also concerned about developing multidrug-resistant *P. aeruginosa*. We also expected that the healthy and thick granulation tissue would localize and completely isolate persistent *P. aeruginosa*, which we believe did not directly cause the severe PPE with foul-smelling pus at the onset of PPE with BPF but did colonize the thoracic cavity after OWT was performed. *P. aeruginosa* is an opportunistic pathogen. When performing omentopexy and simple chest closure with persistent *P. aeruginosa* in the thoracic cavity, we only administered cefazoline to prevent surgical site infection, not covering *P. aeruginosa.* This is because we considered that the intravenous administration of antibiotics covering *P. aeruginosa* would not eradicate *P. aeruginosa* in the thoracic cavity, and we were concerned about developing multidrug-resistant *P. aeruginosa*. Even when performing omentopexy with thoracic and abdominal cavity connection by using the diaphragm tunneling method, there were no perioperative complications caused by *P. aeruginosa* observed.

The long-term outcome of simple chest closure is unclear especially when combined with systemic therapy for lung cancer. However, accepting the presence of the residual pleural cavity may be an option for chest closure, provided that the space is covered with healthy granulation tissue and no BPF is noted. The successful outcome of the Clagett procedure may depend on healthy granulation, which could isolate the empyema, rather than antibiotic filling, especially when the main organism is persistent *P. aeruginosa*.

Patients with recurrent cancer could be candidates of chest closure. These patients were not supposed to be indicated for definitive chest closure using conventional techniques, and the absence of recurrent disease was one of the criteria for OWT closure [1, 4, 6, 7, 12, 13]. Simple chest closure is less invasive compared to thoracoplasty or thoracomyoplasty, avoiding resection of several ribs. Recurrent cancer cases could be candidates of chest closure performed with less invasive techniques.

## Conclusion

We can close a thoracostomy using a simple chest closure technique, despite the presence of the residual space, provided that no BPF is noted and healthy granulation tissue covers the whole cavity. This technique allows careful observation of the “silent empyema,” is less invasive, and can be applied to recurrent cancer cases.

## Abbreviations

BPF: Bronchopleural fistulae
OWT: Open-window thoracostomy
POD: Postoperative day
PPE: Postpneumonectomy empyema
